# A case of stercoral colitis with marked elevation of serum carcinoembryonic antigen

**DOI:** 10.1002/ccr3.2739

**Published:** 2020-02-15

**Authors:** Kiyoto Takehara, Yuko Takehara, Satoshi Ueyama, Tatsunori Kobayashi

**Affiliations:** ^1^ Department of Surgery Japanese Red Cross Mihara Hospital Hiroshima Japan; ^2^ Department of Gastroenterological Surgery Japanese Red Cross Okayama Hospital Okayama Japan; ^3^ Department of Surgery Okayama City Hospital Okayama Japan

**Keywords:** carcinoembryonic antigen, fecal impaction, stercoral colitis

## Abstract

It should be noted that the serum CEA level can become elevated in severe stercoral colitis. Marked elevation of the serum CEA level in stercoral colitis may suggest the necessity of surgery in patients with stercoral colitis.

## Graphical abstract

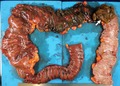

## 1 INTRODUCTION

We report a case of stercoral colitis with markedly elevated serum carcinoembryonic antigen (CEA) levels. It should be noted that the serum CEA level can become elevated in severe stercoral colitis. Marked elevation of the serum CEA level may suggest the necessity of surgery in patients with stercoral colitis.

Stercoral colitis is an inflammatory condition of the colon caused by fecal impaction. The impacted feces increase intraluminal pressure, leading to ischemia of the bowel that can result in ulceration and subsequent intestinal perforation or ischemic colitis.^1^, ^2^, ^3^ A history of chronic constipation is a known risk factor for stercoral colitis, and the mortality rate in perforated cases is reported to be 32%‐57%.^1^ Immediate surgery should be considered in perforated cases and sometimes in nonperforated cases because they can be complicated by ischemic colitis, which can lead to perforation, peritonitis, septic shock, and eventual death if not treated promptly.^2^

Carcinoembryonic antigen (CEA) is a widely used tumor marker for colorectal cancer and various other types of cancer. Serum CEA levels can also be increased in various nonmalignant conditions, including gastrointestinal disorders such as inflammatory bowel disease,^3^, ^4^, ^5^, ^6^ and it is reported that ulcerative colitis activity is correlated with serum CEA levels.^3^ However, the elevation of serum CEA levels in patients with stercoral colitis has not previously been reported in the English literature.

## 2 CASE PRESENTATION

**Figure 1:**
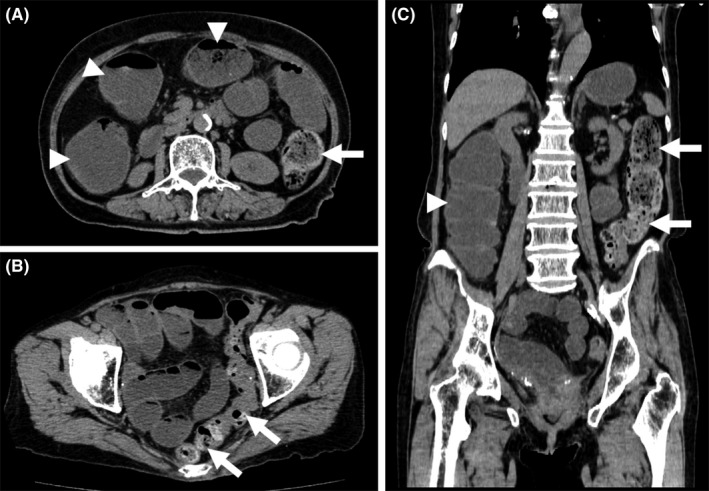
Computed tomography. Axial (A, B) and coronal (C) views show fecal impaction in the left colon and rectum (arrows) and dilatation of the oral side of the colon (arrowheads)

An 80‐year‐old female who had a history of colon diverticulitis and chronic constipation presented with abdominal pain, vomiting, and diarrhea. At the time of admission, her temperature was 36.4°C, blood pressure was 136/66 mm Hg, pulse rate was 73/min, and respiration rate was 20/min. Physical examination revealed tenderness in her left lower abdomen. Bowel sounds were slightly increased. Muscular defense in the abdomen was not obvious. The results of laboratory tests were as follows: leukocyte count, 14 600/µL, with 80.8% neutrophils; lactate dehydrogenase, 364 IU/l; C‐reactive protein, 5.75 mg/dL; creatinine, 0.95 mg/dL; procalcitonin, ≥10.0 ng/mL; lactate, 60 mEq/L; and CEA, 1240.9 ng/mL. Computed tomography revealed marked fecal impaction in the left colon and rectum, with dilatation of the oral side of the colon (Figure 1). Neither extraluminal gas nor fluid collection was observed.

**Figure 2:**
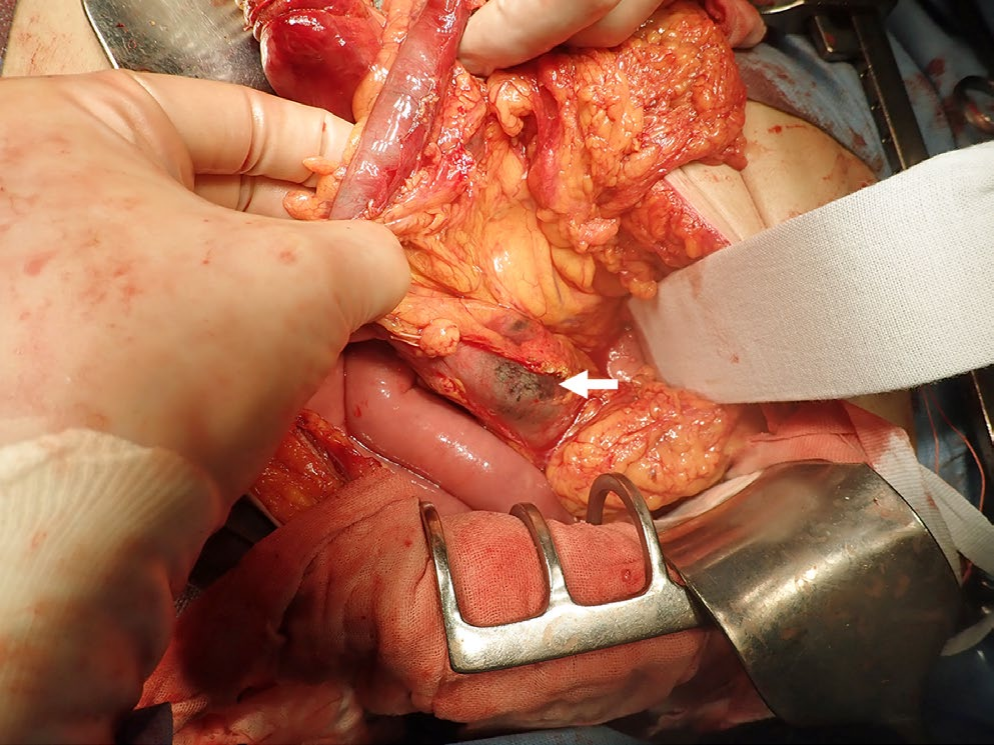
Intraoperative findings. The serous surface of the colon shows a grayish necrotic color (arrow)

**Figure 3:**
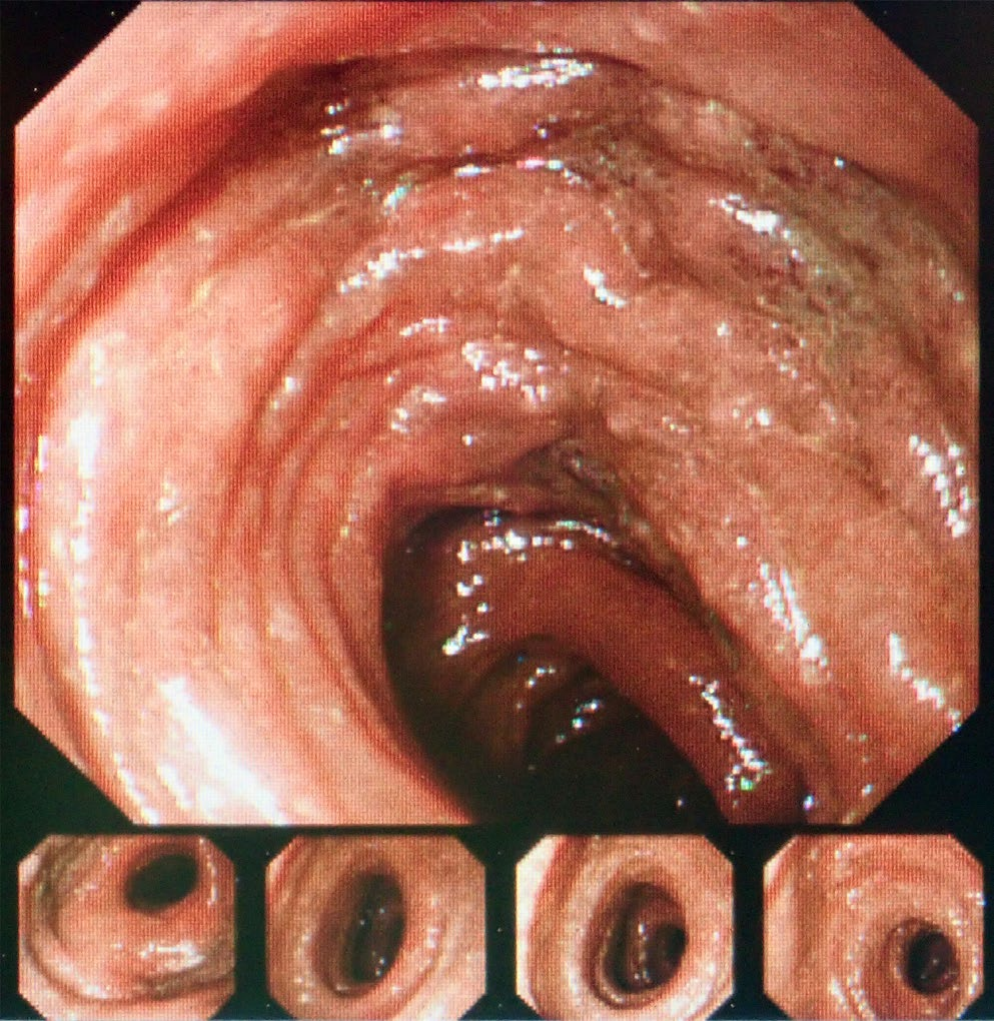
Intraoperative endoscopic findings. The mucosa of the distal ileum was necrotized

**Figure 4:**
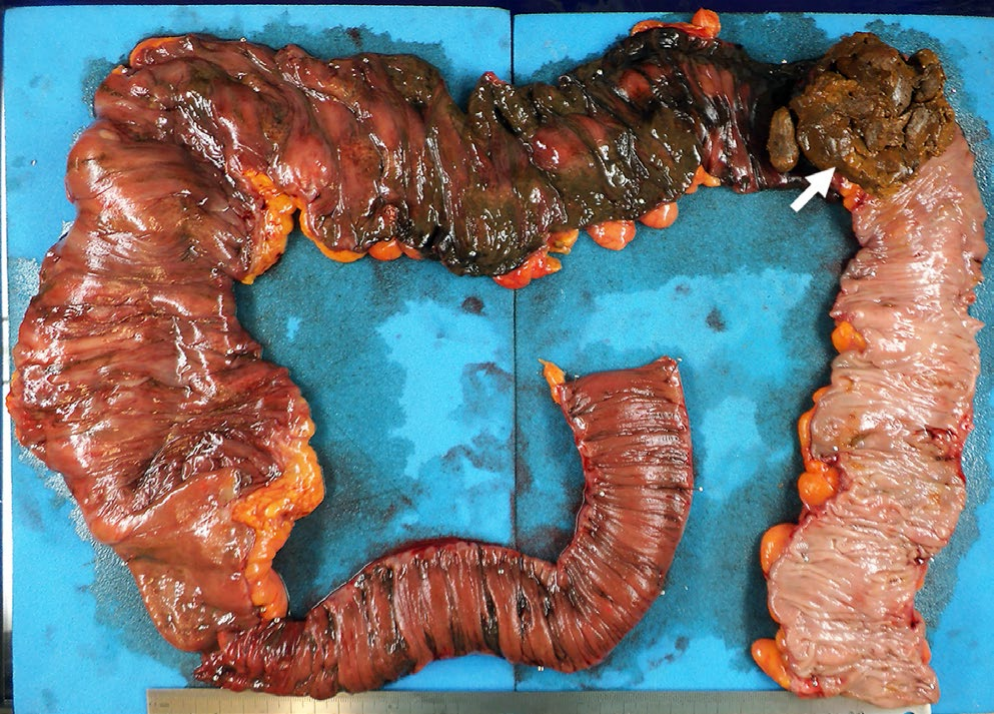
Resected specimen. A fecal mass (arrow) obstructed the descending colon, and the proximal bowel was widely necrotic

**Figure 5:**
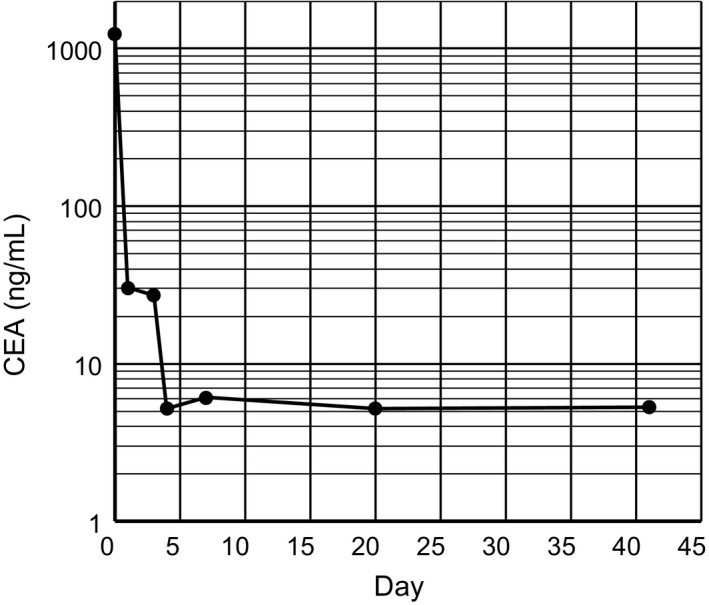
Time course of the serum CEA level

Emergency operation was performed for the diagnosis of severe stercoral colitis. Lower laparotomy was performed. The proximal descending colon was obstructed by a fecal mass, and the proximal side of the colon was necrotic (Figure 2). Multiple diverticula were found in the descending colon. Extensive colectomy was performed, and the oral side was resected at the terminal ileum and anal side at the sigmoid‐descending colon junction using linear staplers. Intraoperative endoscopy from the oral stump of the ileum revealed mucosal necrosis of the distal ileum (Figure 3). The distal ileum was additionally resected, and ileostomy was performed. In the resected specimen, a fecal mass obstructed the descending colon, and the mucosa of the proximal bowel was widely necrotized (Figure 4). The serous surface of the additional resected distal ileum was intact. Histopathological study indicated no malignancy in the resected specimen. Serum CEA levels markedly decreased with time after surgery (Figure 5). The patient survived the operation and remained well 18 months after surgery.

## 3 DISCUSSION

Stercoral colitis is mostly seen in elderly individuals and is more common in old females. It is related to patients being bedridden or having neuropsychiatric disorders, with a poor performance status and a history of chronic constipation along with other comorbidities.^7^, ^8^, ^9^ Patients with stercoral colitis usually present with abdominal pain and sometimes with distention, nausea, vomiting, melena, or diarrhea.^2^, ^8^, ^9^ Most patients have elevated white blood cell counts with a shift to the left. Moreover, there may be signs of peritoneal irritation or septic state; however, physical examination or laboratory tests are not always reliable for diagnosing stercoral colitis.^9^

Carcinoembryonic antigen (CEA), a glycoprotein with a molecular weight of approximately 180 kDa, was initially described by Gold and Freedman in 1965.^10^, ^11^ CEA is widely expressed on the surface of tumor cells of various human tissues as well as on the epithelial cells of normal gastrointestinal tissues and fetal intestine.^12^ Although CEA mRNA is expressed as actively in normal colon mucosa as in cancer tissues, normal colon mucosa contains a very small quantity of CEA, and the concentration of CEA in normal serum is very low.^13^ In the normal colon, single‐layered columnar epithelial cells express CEA on the apical surface of cell membranes facing the lumen and rapidly release it into the lumen of the digestive tract, such that CEA does not directly flow into blood capillaries. In contrast, in cancer tissues that have lost single‐layer organization, neoplastic cells located deep inside tumor glands express CEA on all sides of the cell surface directly facing the blood vessels.^12^, ^14^

Smithson et al reported that CEA is upregulated in inflamed human intestinal tissues.^15^ Sugarbaker reported markedly elevated serum CEA levels in patients with acute colonic obstruction from colorectal cancer.^16^ Bowel decompressive procedures prior to tumor resection markedly reduce serum CEA levels, which suggests that colonic obstruction is independently associated with the elevation of serum CEA levels. Serum CEA levels are rarely greater than 10 ng/mL in benign disorders,^3^ and markedly elevated serum CEA levels in patients with colonic obstruction from nonmalignant causes are rarely reported; one of the reasons for this may be that serum CEA levels are rarely measured in benign conditions.

Moreover, when the elevation of serum CEA levels is observed in nonmalignant patients, the existence of cross‐reacting antigen with CEA should be considered. The major members of the CEA family antigens include nonspecific cross‐reacting antigen (NCA) identified in normal human lung and spleen 17, 18; NCA‐2 in meconium 19; normal fecal antigen‐1 (NFA‐1), NFA‐2 and normal fecal cross‐reacting antigen (NFCA) in normal adult feces 20, 21; and biliary glycoprotein‐1 (BGP‐1) in normal bile.^22^ Since NCA‐2 and NFA‐2 have been found to be the same gene products as CEA, they are considered to be normal counterparts of CEA produced by colon epithelial cells of fetuses and by those of normal adults, respectively.^23^ The cross‐reactivity of commercially available enzyme immunoassay (EIA) kits for CEA differs depending on the products.^24^ According to the manufacturers' data, the EIA kits used in our hospital are reactive with NCA‐2 but not with NCA. Unfortunately, there are no data on reactivity with NFAs. While the half‐life of CEA in blood is reported to be 4‐12 days,^25^, ^26^, ^27^ the rate of decrease in serum CEA levels in our case was clearly faster than that. Although the half‐life times of NFAs are unknown, this finding suggests the possibility of the involvement of NFAs in serum CEA elevation if the EIA kits show cross‐reactivity with NFAs.

Considering these findings, we hypothesized that CEA overexpression caused by bowel inflammation might remain in the intestinal lumen due to fecal colonic obstruction and translocate into blood vessels through damaged intestinal mucosa, resulting in the marked elevation of serum CEA levels in our case. In addition, CEA‐related antigens in feces might be involved in serum CEA elevation.

Stercoral colitis often lacks peritoneal irritation symptoms and CT findings of peritonitis, especially in nonperforated cases, which sometimes makes the decision for surgery difficult. However, the delay of surgery can result in fatal outcomes. Therefore, comprehensive judgment is needed for the decision to perform surgery. In addition, marked elevation of serum CEA levels may suggest the severity of stercoral colitis. If computed tomography rules out colorectal cancer and reveals stool impaction, markedly elevated CEA levels may suggest the severe condition of stercoral colitis and may help to determine the indication for surgery.

## CONFLICT OF INTEREST

The authors declare that they have no conflicts of interest.

## AUTHOR CONTRIBUTIONS

KT: conceived and designed, analyzed and interpreted the data, drafted the article, critically revised the article for important intellectual content, and approved the final manuscript. YT and SU: analysed and interpreted the data and approved the final manuscript. TK: conceived and designed the study and approved the final manuscript.
